# Macromolecular Architecture-Directed Crystallization: Heterogeneous and Homogeneous Crystallization in Miktoarm Star Copolymers

**DOI:** 10.1021/acs.macromol.5c02743

**Published:** 2025-11-28

**Authors:** Dimitrios Chatzogiannakis, Emmanouil Mygiakis, Martin Dulle, Emmanuel Stiakakis, Georgios Sakellariou, Emmanouil Glynos

**Affiliations:** † 37777 Institute of Electronic Structure and Laser, Foundation for Research and Technology-Hellas, P.O. Box 1385, Heraklion, Crete 711 10, Greece; ‡ Department of Chemistry, University of Crete, P.O. Box 2208, Heraklion, Crete 710 03, Greece; § Department of Chemistry, 68993 National and Kapodistrian University of Athens, Panepistimiopolis Zografrou, Athens 15 771 Greece; ∥ JCNS-1 Neutron Scattering and Soft Matter, Forschungszentrum Jülich, Jülich D-52425, Germany; ⊥ Biomacromolecular System and Processes, Institute of Biological Information Processing (IBI-4), 28334 Forschungszentrum Jülich, Jülich D-52425, Germany; # Department of Materials Science and Technology, University of Crete, Heraklion 71003, Greece

## Abstract

Understanding how polymer architecture affects crystallization behavior is crucial for developing advanced functional materials. In this study, we investigate a series of miktoarm star-shaped copolymers composed of polystyrene (PS) and poly­(ethylene oxide) (PEO), with systematically varied PEO content (18–86 wt %) and a fixed PS arm length. Using differential scanning calorimetry (DSC), polarized optical microscopy (POM), and small- and wide-angle X-ray scattering (SAXS/WAXS), we elucidate how intramolecular confinement within star-shaped architectures influences crystallization. At high PEO content, a core–shell morphology supports heterogeneous nucleation and bulk-like spherulitic growth. As PEO content decreases, homogeneous nucleation becomes dominant, and crystallinity is significantly suppressed due to increased confinement and reduced domain size. Our results demonstrate that molecular architecture profoundly influences the nucleation mechanism, crystallization temperature, and crystalline domain structure in PS–PEO miktoarm copolymers, offering a platform for tuning various properties of polymer materials.

## Graphical abstract

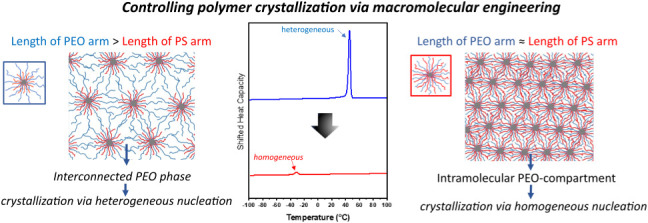

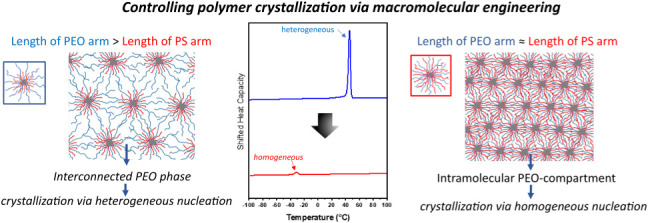

## Introduction

The subject of polymer crystallization has been central to polymer science for decades, as the degree of crystallinity significantly impacts the material’s mechanical, ionic conductivity, and optical transparency.^1,2^ In bulk polymers, crystallization is typically initiated through heterogeneous nucleation, where defects within the material act as nuclei for crystal growth. However, crystallization may also occur via surface nucleation at dissimilar interfaces or through homogeneous nucleation (self-nucleation), an intrinsic, spontaneous process. Heterogeneous nucleation generally occurs over a narrow temperature range above the glass transition temperature (*T*_g_), and appears as a single exothermic peak in differential scanning calorimetry (DSC) during cooling from the melt.

However, when a semicrystalline polymer is confined into microdomains smaller than the critical volume required for bulk nucleation, i.e., smaller than per nucleus in the bulk, multiple independent crystallization events may occur, a phenomenon known as fractionated crystallization. Amorphous-semicrystalline linear block copolymers (BCPs) serve as ideal model systems for studying crystallization in confined geometries.^3–8^ The degree and type of confinement can be systematically adjusted and tuned by modifying the block composition. Depending on the *T*_g_ of the amorphous block relative to the crystallization temperature (*T*_c_) of the semicrystalline block, the confinement can be categorized as either hard (*T*_g_ of the amorphous block > *T*_c_ of the semicrystalline block) or soft (*T*_g_ of the amorphous block < *T*_c_ of the semicrystalline block). Various BCP systems have been investigated in the literature, including PE-*b*-PS,^3^ PB-*b*-PEO,^4^ PCL-*b*-PS,^5,6^ PE-*b*-PVCH,^7^ or PS-*b*-PEO.^8^

Linear poly­(ethylene oxide), PEO, homopolymer crystallizes around 45 °C via heterogeneous nucleation, presenting a single exothermic peak in DSC. In PS-*b*-PEO systems with a lamellar morphology, PEO crystallizes under hard confinement (*T*_g_ of PS > *T*_C_ of PEO) and behaves similarly to bulk PEO (heterogeneous nucleation) due to the defects present within the lamellae. In contrast, when PEO is confined within cylindrical domains in a PS matrix, crystallization shifts to homogeneous nucleation. In such systems, the number of PEO cylinders is several orders of magnitude greater than the number of nucleating defects, rendering the cylinders statistically free of impurities and enabling homogeneous nucleation at higher supercooling (that is, the difference between the apparent melting temperature, *T*_m_^′^ and apparent crystallization temperature, *T*_c_^′^).

Fractionated crystallization has also been observed in semicrystalline polymers confined in well-ordered anodic aluminum oxide (AAO) templates. Floudas and coworkers showed that while bulk PEO crystallizes via heterogeneous nucleation, PEO confined in AAO pores smaller than 65 nm undergoes homogeneous nucleation.^9^ In these systems, only a small portion of pores contain nucleating defects, and the compartment volumes are too small to support typical bulk nucleation, leading to crystallization at higher supercooling.

Beyond linear BCPs, nonlinear copolymers—particularly star-shaped macromolecules—offer new opportunities for studying polymer crystallization under confinement. Star polymers, comprising multiple linear chains (arms) grafted onto a central core, exhibit properties that are intermediate between those of linear chains and colloid particles.^10–12^ Harmandaris and coworkers showed that miktoarm star copolymers, such as (PS)*_n_* (PEO)_*n*_, where *n* denotes the number of arms, experience intramolecular confinement due to unfavorable interactions between the PS and PEO arms, with their morphology dictated by both the number and length of the arms.^13^ While (PS)*_n_* (PEO)_*n*_ stars with functionalities lower than 16 (i.e., *n* < 8) nanosegregate or phase-separate into two main domains resembling Janus particles, miktoarm stars with 32 arms (*n* = 16) form multipatchy nanoparticles.^13^ Beyond the fundamental interest, these materials are of interest for lithium-ion-conducting solid polymer electrolytes. In previous work, we demonstrated that star-shaped polymers can be engineered to achieve both high modulus and high ionic conductivity, making them promising candidates for advanced energy storage applications.^14–18^

Here, we use poly­(ethylene oxide), PEO, as a model crystallizable polymer to systematically investigate the influence of macromolecular architecture and intramolecular hard confinement on crystallization behavior. Differential scanning calorimetry (DSC), small- and wide-angle X-ray scattering (SAXS/WAXS), and polarized optical microscopy (POM) were employed. A series of miktoarm copolymer stars were synthesized, each comprising >30 PEO arms and an approximately equal number of PS arms. The weight fraction of the PEO was varied from 18% to 86%, and these molecules are referred to as SPSPEO-A-B, where A and B denote the weight percentages of PS and PEO, respectively. The molecular weight of the PS arms was kept constant at approximately 7 kg/mol, ensuring a uniform PS arm length across the series. When PEO arms exceed PS arms in length, a core–shell morphology emerges with a nanostructured PS–PEO core and a crystallizable PEO shell (Figure 1). As the fraction of PEO decreases, the PEO shell thins and ultimately declines when PS and PEO arms are of comparable length. This tunable nanostructure enables a systematic investigation of the effects of macromolecular architecture and confinement on crystallization, with potential applications in advanced materials, including solid polymer electrolytes.

## Experimental Section

### Materials

#### Synthesis of Miktoarm Star-Shaped Copolymers

The miktoarm star-shaped poly­(ethylene oxide)–polystyrene copolymers, SPSEO, with *n* number of arms, were synthesized using the “arm-first” method. This approach involves the sequential anionic polymerization of styrene and divinylbenzene (DVB), leading to the formation of relatively well-defined star-shaped polymers (SPS) with anionically active cores.^19–21^ Subsequently, a second set of PEO arms was grown out from the core by utilizing the living carbanionic sites present in the core. Further details on the synthesis and characterization of these star-shaped copolymer can be found in a recent publication.^21^ The molecular weight of the PS arms was maintained at approximately 7 kg/mol, ensuring uniformity in PS arm length across the series. The wt % of the PEO arms was systematically varied from 18% to 86 wt %, resulting in a range of miktoarm star copolymers—from those with comparable PS and PEO arm lengths to those with progressively longer PEO arms (Figure 1). In this study, these miktoarm stars will be termed SPSPEO-A-B, where A and B denote the weight percentages of PS and PEO, respectively. The molecular characteristics of the star-shaped copolymers under investigation in this study are reported in Table 1.

**Figure 1:**
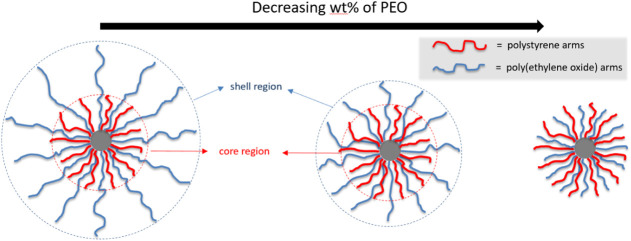
Graphical representation of the molecules studied showing the varying length of the PEO arm (blue arms) and the core–shell-like appearance for high PEO weight fractions when the PEO arms are longer than the PS ones.

**Table 1:** Molecular Characteristics of Miktoarm Star Copolymers PS_n_–PEO_m_

Sample	PS arm M_w_ (kg/mol)^a^	PS_*n*_ star M_w_ (kg/mol)^b^	PS weight fraction (% w/w)^c^	PS_n_–PEO_m_ *M*_w_ (kg/mol)^c^	f PS^d^
SPSEO-14-86	7	1.150	14	3.190	145
SPSEO-21-79	7	1.150	21	2.130	145
SPSEO-43-57	7	1.150	43	1.740	145
SPSEO-65-35	7	1.150	65	1.770	145
SPSEO-82-18	8.7	259	82	350	30

a Determined by GPC in CHCl_3_ at 25 °C.

b Determined by Static Light Scattering in toluene at 25 °C.

c Calculated from ^1^H NMR.

d f: Functionality, number of PS arms.

### Differential Scanning Calorimetry (DSC)

The thermal properties of the samples were analyzed using a Discovery 250 (TA Instruments) DSC. The instrument was calibrated for heat capacity using a sapphire disk and for temperature and enthalpy using indium. To eliminate any prior thermal history, the samples were annealed under a N_2_ atmosphere at 150 °C for 10 min before measurements. Two distinct measurement protocols were employed in this study: (i) A dynamic scan mode in which the temperature was continuously varied at a rate of 10 °C/min following a heat–cool–heat cycle ranging from −110 to 150 °C, and (ii) An isothermal crystallization mode in which the sample was first heated at 150 °C for 10 min and then rapidly cooled at a rate of about 50 °C/min to a desired temperature.

The degree of crystallinity (χ_c_) from the DSC thermographs was determined from the melting enthalpy, normalized to the PEO mass in the sample, using

$$\chi_{c}=\frac{\Delta H_{m}}{\Delta {H_{m}}^{0}}\times 100\%$$

where Δ*H*_m_ is the measured heat of fusion and Δ*H*_m_^0^ = 196.4 J g^–1^ is the heat of fusion of 100% crystalline PEO.

### Polarized Optical Microscopy (POM)

The samples included in this study were also studied by polarized optical microscopy (POM), where the sample placed in a temperature-controlled stage is observed between crossed polarizers. Normally, no light passes through the system, resulting in a dark black image. However, when crystals form, they interfere with the polarized light, causing bright regions to appear in the image and revealing crystalline domains. Since polymers typically crystallize through a nucleation and growth process, POM enables the visualization of spherulite evolution over time, providing insights into crystallization kinetics. In this study, POM was combined with a LabView-based image processing script, which analyzes both the intensity and temperature data, generating intensity vs temperature plots. By integrating these curves, we obtain d*I*/d*T* plots, which reflect the crystallization rate as a function of temperature and are qualitatively comparable to the DSC thermograms (Figure 2).

**Figure 2:**
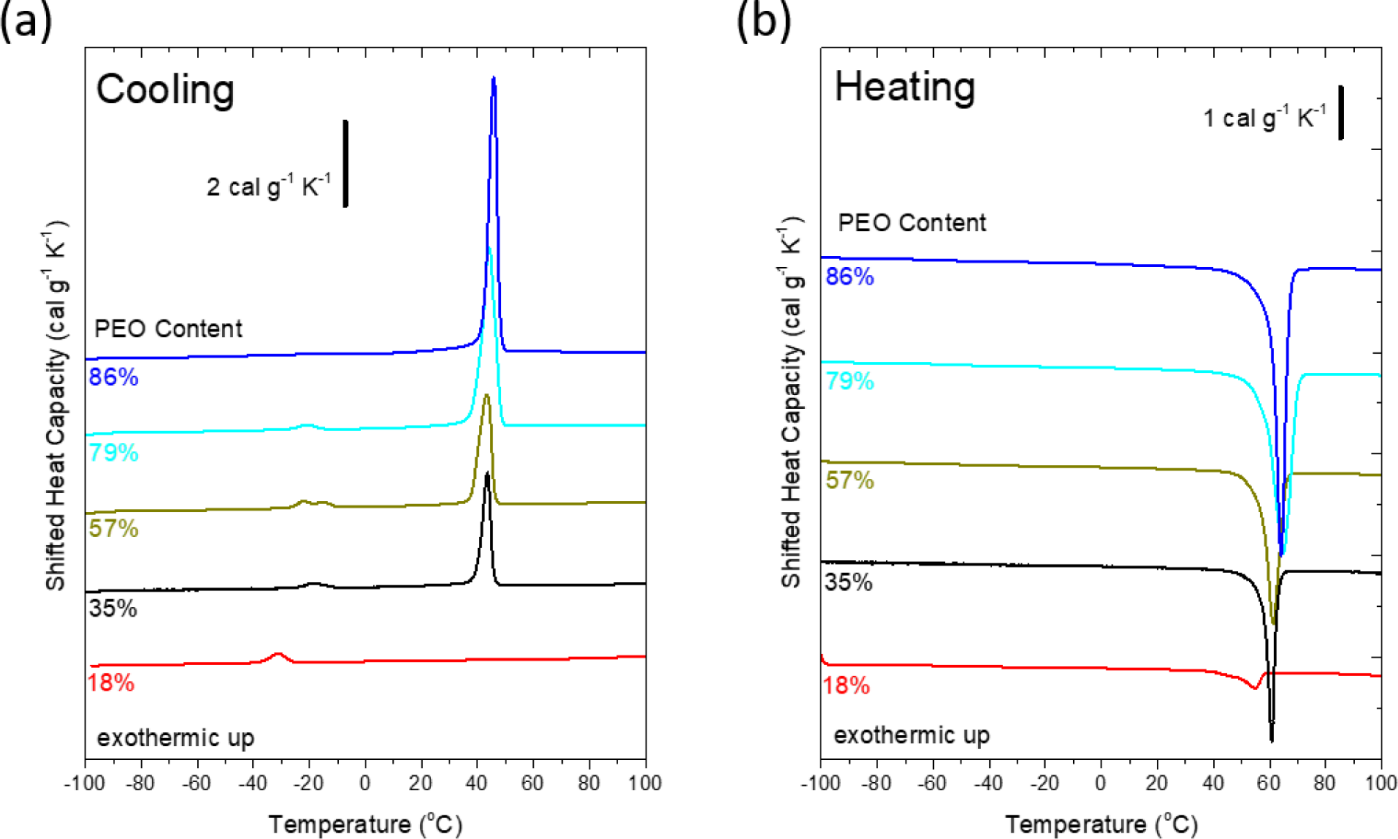
Cooling (a) and subsequent heating (b) DSC thermographs for PS–PEO miktoarm stars: From top to bottom, the curves correspond to SPSEO-14-86 (blue line), SPSEO-21-79 (light blue line), SPSEO-43-57 (dark yellow line), SPSEO-65-35 (black line), and SPSEO-82-18 (red line).

### Small- and Wide-Angle X-ray Scattering (WAXS, SAXS)

The SAXS measurements were conducted using the “Ganesha-Air” system (SAXSLAB, Xenocs), equipped with a D2-MetalJet (Excillum) X-ray source featuring a liquid-metal anode. The source operated at 70 kV and 3.57 mA, emitting Ga-Kα radiation (λ = 0.1341 nm), which provided a highly brilliant and focused beam (<100 μm). A custom-designed X-ray optic (Xenocs) with a 55 cm focal length was used to generate a small, intense beam at the sample position. The large Eiger4M detector enabled a single detector distance of 35 cm to capture both large-scale structures at low *q* and crystalline peaks at high *q*. Samples were mounted on a Linkam TST350 heating/cooling block connected to a liquid nitrogen pump and reservoir. Each sample was first heated to 150 °C to ensure the complete melting of the structure, confirmed by an initial SAXS scan. A series of SAXS measurements was then conducted during slow cooling, with an exposure time of 30 s per frame and a cooling rate of 2 °C/min, resulting in each SAXS measurement corresponding to a Δ*T* of 1 °C. The cooling process spanned from 150 °C to −80 °C, yielding 231 images per sample with virtually no dead time between frames.

2D SAXS images were radially averaged, corrected for transmission, and background-subtracted using an empty TST350 measurement. The degree of crystallinity was estimated by analyzing the high-*q* range (3–17 nm^–1^), where the crystalline peaks are prominent. The amorphous background was fitted for each curve, and the crystalline fraction was defined as the difference between the total signal and the fitted amorphous contribution. The degree of crystallinity was determined as the ratio of the crystalline signal area to the total signal area, as reported in the main text.

## Results and Discussion

The influence of macromolecular architecture and composition on the crystallization behavior of PEO in PS–PEO miktoarm stars was investigated using DSC, POM, and SAXS/WAXS. The weight fraction of the PEO arms was systematically varied from 18 to 86 wt %, producing a series of miktoarm star copolymers ranging from architectures with comparable PS and PEO arm lengths to those containing progressively longer PEO arms (Figure 1). Figure 2 shows DSC cooling (Figure 2a) and heating (Figure 2b) at rates of 10 °C/min for all of the various star copolymers utilized in this work (Table 1). SPSEO-14-86 (blue lines, Figure 2) exhibits a sharp crystallization exotherm with a peak at *T*_c_ = 46 °C and a sharp melting endotherm with a peak at *T*_m_ = 64 °C, which is typical of heterogeneously nucleated bulk PEO. The corresponding POM image (Figure 3) reveals the formation of large, well-defined spherulites during nonisothermal crystallization. As the wt % of PEO decreases to 79% (SPSEO-21-79, light blue lines in Figure 2), the heterogeneous nucleation peak broadens and slightly shifts to *T*_c_ = 44 °C, while a second, weaker exothermic peak emerges at about −21 °C. Further decreasing the PEO content, i.e., for SPSEO-43-57 and SPSEO-65-35 (dark yellow and black lines in Figure 2, respectively), progressively weakens the high-temperature exothermic peak, while the low-temperature exothermic peak remains relatively unchanged. Notably, in the most PS-rich sample of SPSEO-82-18, where PS and PEO arms are similar in length, the high-temperature heterogeneous crystallization feature is absent, and a single low-temperature event is observed at approximately −32 °C, indicating homogeneous nucleation.

**Figure 3:**
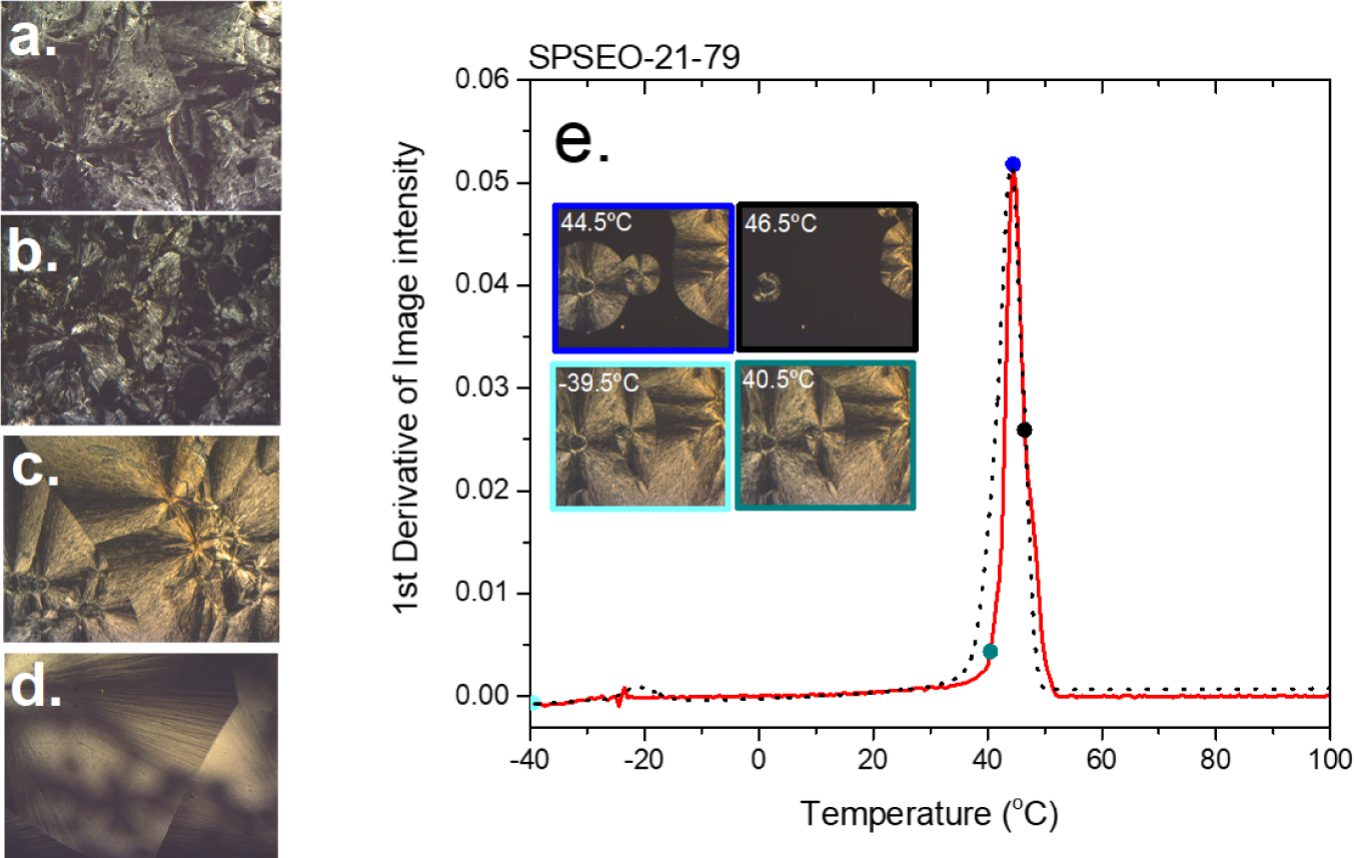
POM images of (a) SPSEO-65-35, (b) SPSEO-43-57, (c) SPSEO-21-79, and (d) SPSEO-14-86. (e) Intensity (red) and DSC (dashed) traces for SPSEO-21-79.

These observations can be rationalized in terms of the molecular architecture. In SPSEO-14-86, the polymer particles adopt a core–shell morphology (Figure 1) with a nanostructured PS/PEO core and a PEO-rich shell. This shell forms an interconnected PEO network between particles (miktoarm stars), enabling heterogeneous nucleation and the growth of larger spherulites (as confirmed by POM, Figure 3e). As the PEO arm length decreases, the interconnected shell diminishes, reducing the contribution from heterogeneous nucleation and revealing a secondary crystallization event from PEO domains confined within the core region; POM reveals smaller and ill-defined spherulites. In SPSEO-82-18, which lacks the PEO shell as PEO and PS arms are comparable in length and a distinct PEO shell is absent, PEO crystallization is processed entirely by homogeneous nucleation; POM confirms the absence of spherulites even at −40 °C. The transition from an interconnected PEO corona to discrete intramolecular PEO compartments with decreasing PEO arm length is schematically illustrated in Figure 4, highlighting how increasing architectural confinement suppresses heterogeneous nucleation and favors homogeneous crystallization.

**Figure 4:**
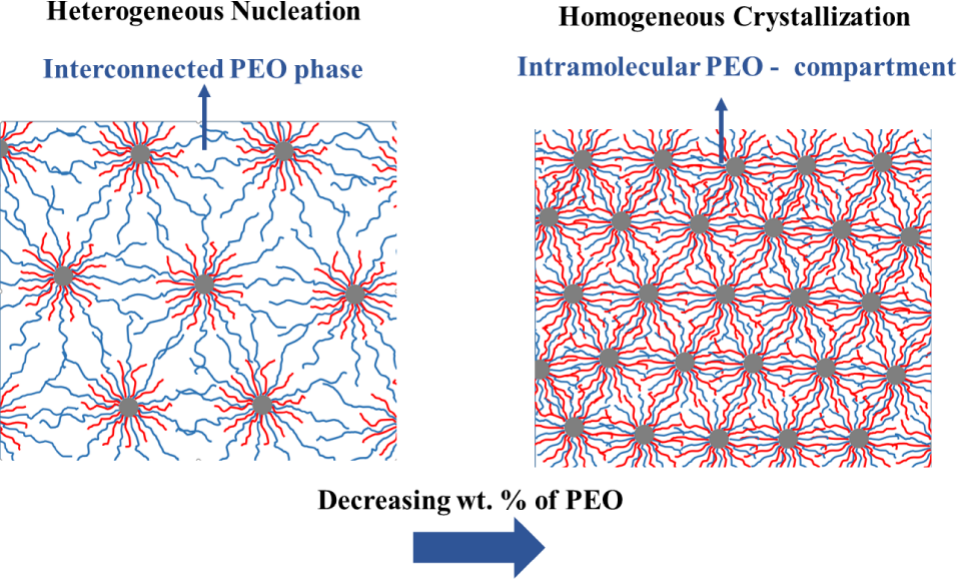
Schematic representation of the transition from an interconnected PEO phase (left) to discrete intramolecular PEO compartments (right) with decreasing PEO arm length in PS-PEO miktoarm star copolymers. This structural change eliminates interdomain connectivity, restricting crystallization to isolated domains and driving the transition from heterogeneous to homogeneous nucleation.

An important distinction must be made between homogeneous nucleation occurring near adsorbing and repulsive interfaces. In systems with weakly adsorbing surfaces, chain flattening and loss of conformational entropy can promote surface-induced nucleation through an entropic effect.^22^ However, when the interface is strongly adsorbing, the enthalpic penalty associated with restricted chain mobility suppresses nucleation entirely, leading to the formation of immobilized amorphous layers.^23,24^ In contrast, the PS/PEO interfaces in the present miktoarm star systems are repulsive due to the inherent immiscibility of the two blocks. These nonadsorbing interfaces do not facilitate surface-induced nucleation; instead, they effectively isolate the PEO domains from external heterogeneities, creating nearly defect-free microenvironments. As a result, crystallization can proceed only through homogeneous nucleation within these confined domains at large supercoolings. This mechanistic distinction highlights that in the case of repulsive interfaces, homogeneous nucleation arises not from interfacial adsorption but from the statistical absence of heterogeneous nuclei.

To further explore crystallization behavior, SAXS/WAXS measurements were performed during cooling from 150 to −80 °C to probe the structural evolution during crystallization. The degree of crystallinity during cooling, estimated from high-*q* SAXS, is plotted as a function of temperature in Figure 5. SPSEO-14-86 exhibits a single crystallization at ∼50 °C. SPSEO-21-79, SPSEO-43-57, and SPSEO-65-35 each display two distinct crystallization events: first one at a high-temperature near 50 °C and second one at a lower temperature around −25 °C, corresponding to heterogeneous and homogeneous nucleation, respectively. SPSEO-82-18 shows only the low-temperature event at about −32 °C. The degree of crystallinity from the WAXS profile is nearly identical for SPSEO-14-86 and SPSEO-21-79 (within the experimental error) and then decreases with decreasing PEO wt %.

**Figure 5:**
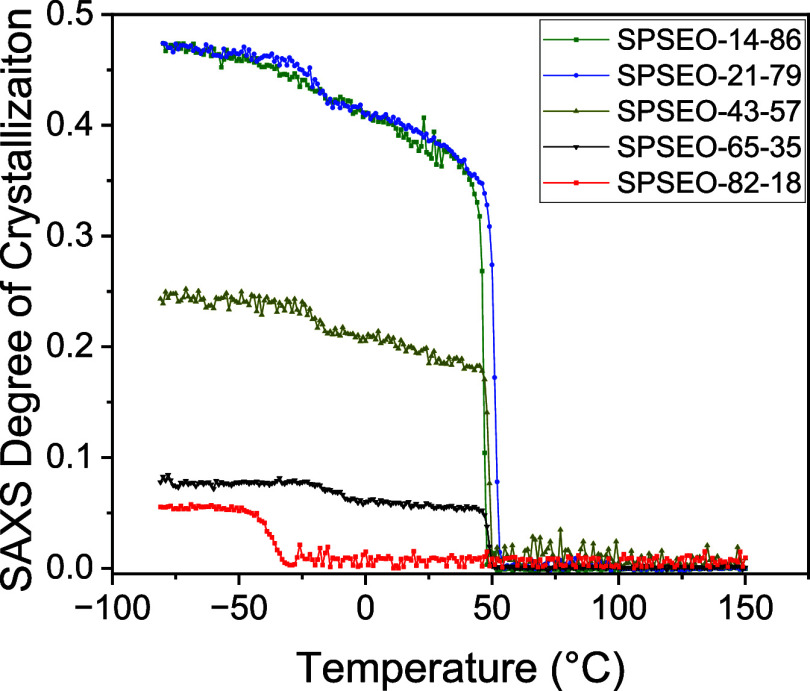
Degree of crystallinity as measured from WAXS measurement during cooling for SPSEO-14-86 (blue dots), SPSEO-21-79 (light-blue dots), SPSEO-43-57 (light-red dots), and SPSEO-65-35 (red dots).

SAXS/WAXS curves for SPSPEO-43-57 at 150 °C (red curve, melt), −10 °C (yellow curve, intermediate), and −80 °C (green curve, fully crystallized) are plotted in Figure 6. At 150 °C, the low-*q* region shows a featureless power-law decay, indicating a homogeneous melt with no contrast-generating structures on the probe length scales. Upon cooling at −10 °C, contrast appears due to crystallization of the outer PEO shell, producing form factor fringes (black arrows). At −80 °C, inner core crystallization eliminates contrast, and the signal becomes featureless. This behavior supports the conclusion that contrast changes—and thus the visibility of the cores—are governed by the crystallization state of both outer and inner PEO domains.

**Figure 6:**
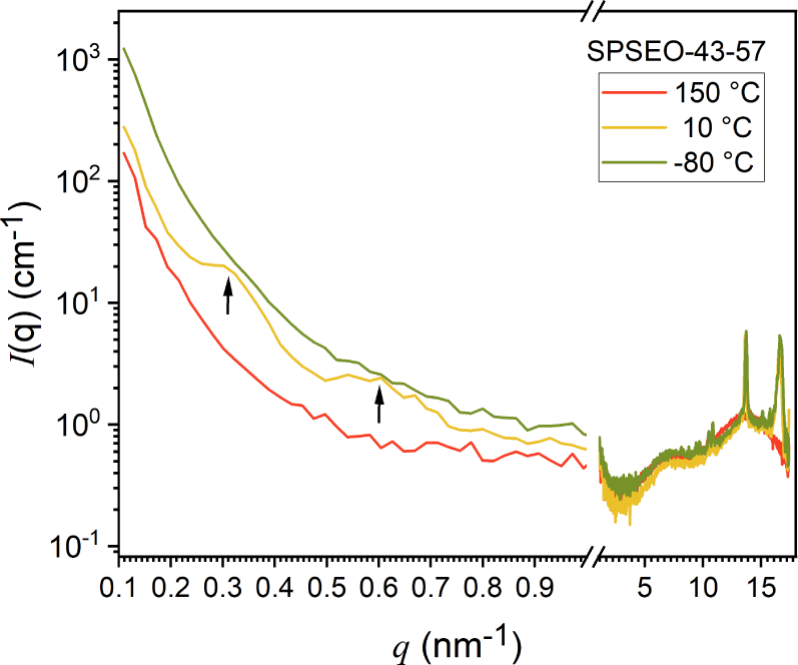
SAXS/WAXS curves of SPSPEO-43-57 at 150 °C (red), 10 °C (yellow), and −80 °C (green).

At −10 °C, the fringes observed in the SAXS data (indicated by black arrows) are attributed to the form factor of the star-shaped cores and were fitted by using a model combining homogeneous spheres with a hard-sphere structure factor. The extracted core radii ranged from 9.5 to 11 nm for all the samples, while the core volume fraction increased as PEO content decreased (see Table 2). Importantly, the core radius remains consistent across all compositions, as expected due to the uniform PS arm length and the corresponding polymerization process. After homogeneous crystallization, form factor fringes vanish, and contrast is lost. Even in SPSEO-14-86, a minor contribution from homogeneously nucleated core domains is observed at intermediate temperatures but is undetectable in WAXS and DSC.

**Table 2:** Fit Values for the Hard Sphere Structure Factor and Spherical Form Factor at *T* = −10 °C

Sample	Excess PEO	Volume Fraction of the Core Region	Radius (nm)	HSR
SPSEO-14-86	0.68	0.31	11	11.1
SPSEO-21-79	0.61	0.405	11	11.4
SPSEO-43-57	0.39	0.47	9.66	9.7
SPSEO-65-35	0.17	0.49	9.5	9.6
SPSEO-82-18	0	-	-	-

The WAXS profiles of the star-shaped SPSPEO copolymers at −80 °C reveal a pronounced dependence of crystallization behavior and order on the PEO content and arm length, which are intricately linked to the topological constraints and resulting nanostructured morphology imposed by the molecular architecture and composition (Figure 7). The scattering patterns for SPSEO-14-86 exhibit strong, well-defined Bragg reflections at 13.6 nm^–1^ and 16.5 nm^–1^, which correspond to (120) and (032) reflections of the orthorhombic lattice of crystalline PEO. These reflections are characteristic of extended chain folding and efficient lamellar stacking in PEO.^25^ To make this point clearer, it is emphasized that all of the diffraction peaks arise exclusively from the crystalline PEO domains. The polystyrene (PS) segments remain completely amorphous throughout the temperature range examined, and no additional reflections corresponding to PS or any other crystalline phase were detected. The absence of extra diffraction features confirms that the crystallization originates solely from the PEO component of the miktoarm stars. This assignment is consistent with previous reports of orthorhombic PEO crystallization.^1,25^

**Figure 7:**
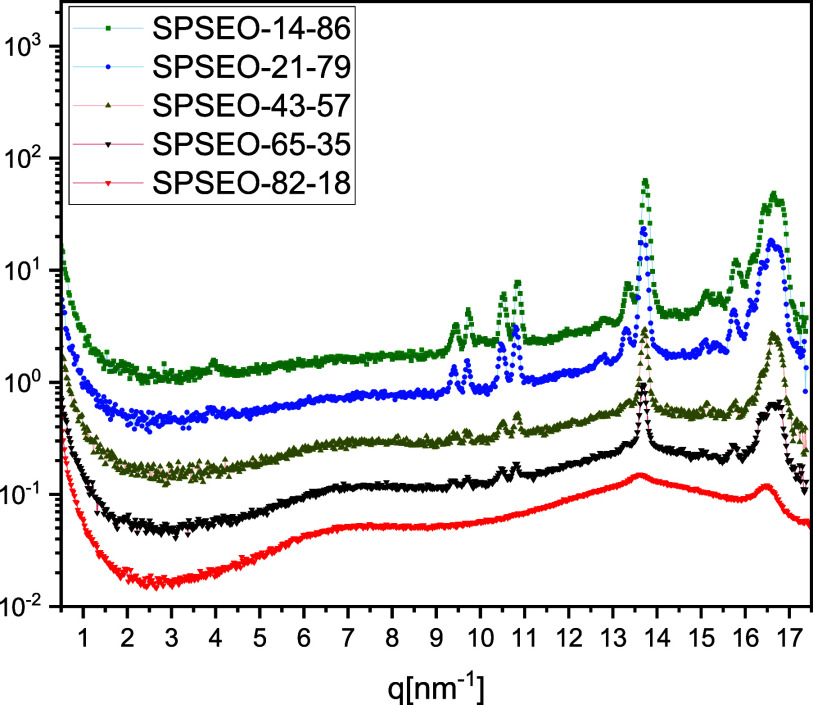
WAXS data at −80 °C for SPSEO-14-86, SPSEO-21-79, SPSEO-43-57, SPSEO-65-35, and SPSEO-82-18.

As the PEO content decreases in SPSPEO‑65‑35, a less ordered structure is observed. The crystalline peaks persist but appear broader and of reduced intensity, indicating either a decrease in the average crystallite size or an increase in lattice disorder. This trend implies that the reduction in the PEO arm length imposes a geometric limitation on the extent of crystallization. While some degree of ordering is preserved, the crystallization process becomes increasingly constrained by the branched core and by steric hindrance from adjacent arms, domains, and PS segments. In SPSPEO-82-18, the most PS-rich sample with the shortest PEO arms, the crystalline peaks are nearly absent, and the scattering pattern is dominated by a broad amorphous halo. This indicates a near-complete suppression of the long-range crystalline order. The combination of short PEO chains likely below the critical length for nucleation and high interfacial curvature arising from the star-like architecture significantly disrupts the ability of PEO segments to adopt the regular chain packing required for crystallization. Taken together, these results demonstrate that crystallization in star-shaped PEO–PS copolymers is governed by a delicate interplay among chain length, architectural confinement, and phase incompatibility. While longer PEO arms can partially overcome topological frustration and crystallize, shorter arms are sterically and energetically hindered from achieving the regular ordering needed to produce detectable crystalline reflections in WAXS.

The DSC traces in Figure 1a show in all cases a single endothermic peak with a monotonic increase in the apparent melting temperature *T*_m_ as the wt % of PEO increases (Figure 8, red points, right *y*-axis). The *T*_m_ of the PEO crystals formed in SPSEO-82-18, i.e., with the lowest wt % of PEO, is about 9 °C lower than that of SPSEO-14-86 that crystallized exclusively via bulk heterogeneous nucleation. The systematic decrease in the melting temperature (*T*_m_) with a decreasing PEO content reflects the combined effects of molecular weight and confinement. According to the Gibbs–Thomson relationship, shorter PEO chains form thinner lamellae with reduced thermodynamic stability, resulting in lower *T*_m_. In addition, the higher concentration of chain ends in low-molecular-weight PEO arms disrupts chain folding and crystalline order. However, in the present miktoarm star systems, this intrinsic chain-length dependence is strongly amplified by the topological confinement imposed by the star architecture. When the PEO arms are longer than the PS arms, an interconnected PEO corona forms (see also Figure 4), enabling lamellar thickening and crystallization behavior similar to bulk PEO. As the PEO arms shorten, this interconnected corona disappears, and the PEO becomes restricted to discrete intramolecular compartments within the PS-rich matrix (see also Figure 4), with characteristic dimensions on the order of the star diameter. In these highly confined stars, crystallization can proceed only within isolated PEO domains via homogeneous nucleation at large supercoolings, leading to thin, imperfect lamellae and a pronounced depression in *T*_m_. This interpretation is consistent with previous studies of confined or tethered PEO systems.^23,26^ Notably, the decrease in *T*_m_ occurs gradually while decreasing the wt % of PEO.

The degree of crystallinity, calculated based on the heat of fusion of 100% crystalline PEO *T*_m_^0^ = 196.4 J/g, strongly depends on the wt % of PEO (Figure 8, black symbols, left *y*-axis), with the SPSEO-82-18 exhibiting a crystallinity as low as χ_c_ = 5%. Interestingly, for 79 and 86 wt % PEO, the degree of crystallinity remains nearly identical within the experimental error, indicating that at high PEO wt %, crystallinity is primarily influenced by the star-shaped architecture of the PEO-based macromolecules rather than the presence of short PS arms. Nevertheless, the star-shaped architecture significantly reduces the degree of crystallinity compared to what is expected for linear PEO chains, as shown by the dashed lines in Figure 8 which indicate the average degree of crystallinity of several linear PEO samples with various molecular weights from 1.9 to 345 kg/mol. This observation aligns with previous reports in the literature.^27,28^ The red line is the average of several linear PEO samples with various molecular weights from 1.9 to 345 kg/mol.

**Figure 8:**
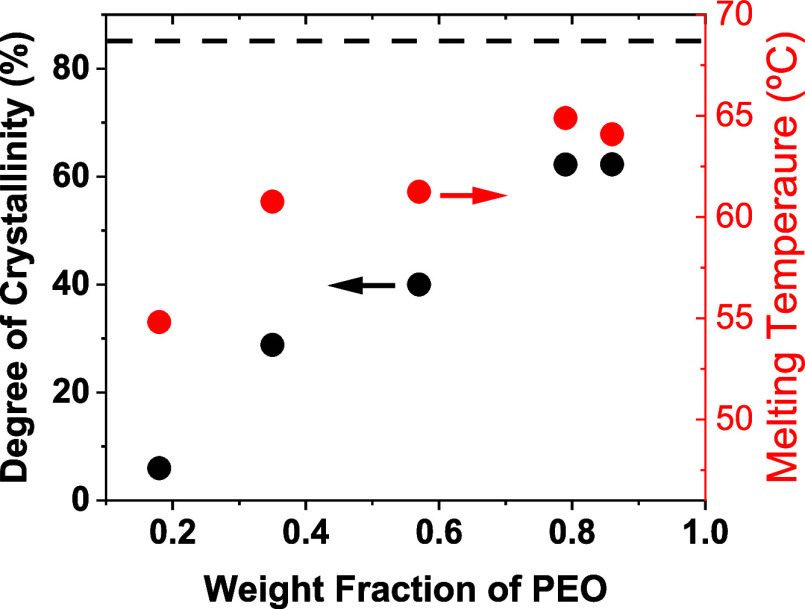
Degree of crystallinity (black points, left *y*-axis) and melting temperature (red points, right *y*-axis) as a function of the wt % of PEO in the miktoarm PS–PEO star copolymers. The black dashed line corresponds to the degree of crystallinity of linear PEO.

Müller and coworkers, by analyzing extensive literature data, established a correlation between the apparent crystallization temperature during a DSC scan, *T*_c_ (in °C), and the void/compartment volume of homogeneously nucleated PEO, *V*_V_ (in nm^3^):^29^

1 $$T_{c}=-41.8+2.89\;\log\left(V_{v}\right)$$

This relationship is based on the premise that the nucleation rate probability is dependent on the segment volume. Based on eq 1, the observed *T*_c_ of −32 °C for the SPSEO-82-18 corresponds to homogeneous nucleation of PEO voids with a volume of about 2460 nm^3^ or to a spherical void with radius *R*_v_ ∼8.5 nm; i.e., void sizes smaller than the *R*_h_ of the SPSEO-82-18 (as estimated from dynamic light scattering (DLS)). This analysis suggests that PEO crystallization occurs intramolecularly, which is expected given that PS and PEO arms have similar lengths. While surface nucleation could be considered as an alternative mechanism, literature evidence suggests that it requires specific macromolecular conformations at the interface and is typically observed in systems dominated by attractive interfacial interactions, such as PEO/alumina nanopores.^30^ In contrast, the PS/PEO interfaces in our study are characterized by repulsive interfacial interactions due to the immiscibility of the involved polymer arms/phases, making homogeneous nucleation the most plausible mechanism.

## Conclusions

In summary, the crystallization pathway of PEO in PS–PEO miktoarm star copolymers is dictated by the relative arm lengths and the resulting phase morphology. Long PEO arms form an interconnected corona that promotes bulk-like heterogeneous nucleation, while shorter arms favor sequential heterogeneous and homogeneous crystallization, and equal PS/PEO arm lengths lead exclusively to homogeneous nucleation at deep supercooling. SAXS/WAXS analyses show constant core radii across compositions but reveal a marked decrease in lamellar order and thickness with reduced PEO content, consistent with intramolecular confinement. These results highlight how architectural design can be used to control crystallization kinetics and morphology in multiphase polymer systems.

## Supplementary Material



## Notes

AXS analysis methodology and crystallinity determination; SAXS hard-sphere modeling and fitting constraints; example fits for samples with short and long PEO arms; data-processing notes and referenced analysis functions (PDF)
