# Supplementary material for: Macromolecular Architecture-Directed Crystallization: Heterogeneous and Homogeneous Crystallization in Miktoarm Star Copolymers
Source: Macromolecules. 2025 Nov 28;58(23):12739–46. doi: 10.1021/acs.macromol.5c02743 (PMC12874644; doi:10.1021/acs.macromol.5c02743)
Supplement: Supplementary file 1 [file ma5c02743_si_001.pdf]

SUPPORTING INFORMATION

Macromolecular Architecture-Directed Crystallization: Heterogeneous and Homogeneous Crystallization in Mikto-arm Star Copolymers

Dimitrios Chatzogiannakis,[†,‡] Emmanouil Mygiakis,[⊥] Martin Dulle,[%] Emmanuel Stiakakis,[#] Georgios Sakellariou,[⊥] and Emmanouil Glynos,[†,±,*]

[†] Institute of Electronic Structure and Laser, Foundation for Research and Technology-Hellas, P.O. Box 1385, 711 10 Heraklion, Crete, Greece

[‡] Department of Chemistry, University of Crete, P.O. Box 2208, 710 03 Heraklion, Crete, Greece

[⊥] Department of Chemistry, National and Kapodistrian University of Athens, Panepistimiopolis Zografrou, 15 771 Athens, Greece

[%] JCNS-1 Neutron Scattering and Soft Matter, Forschungszentrum Jülich, D-52425 Jülich, Germany

[#] Biomacromolecular System and Processes, Institute of Biological Information Processing (IBI-4), Forschungszentrum Jülich, D-52425 Jülich, Germany

[±] Department of Materials Science and Technology, University of Crete, Heraklion 71003, Greece

*Corresponding author: eglynos@iesl.forth.gr , eglynos@mateials.uoc.gr

WAXS Data Analysis

For all WAXS data in the main text, identical data reduction and fitting procedures were applied. The reduction, described in the main text, was performed over the full q range at once. To

determine the degree of crystallinity. The high q range ($3\text{nm}^{-1} \leq q \leq 17\text{nm}^{-1}$) was resampled to 250 points. This range was selected because the intensity between q between 2 and 4 nm^{-1} is essentially featureless and temperature-independent, providing a stable background reference. The upper limit ($q = 17\text{nm}^{-1}$) was chosen to minimize the edge and shadowing effects while capturing the main PEO crystalline peaks. The crystallinity was calculated as the ratio of the crystalline-peak area to the total scattered intensity:

$$X_C = \frac{A_{total} - A_{amorph}}{A_{total}}$$

The amorphous background, nearly temperature independent across all samples, was fitted using the sum of two Voigt functions fitted.[1] Lower bounds were imposed on the peak widths to prevent the crystalline peaks from being absorbed into the background fit. This minimal-parameter model allowed all datasets to be fitted consistently with the same approach. Representative fits for samples with the shortest and longest PEO arms are shown in Figure S1.

Uncertainty in $X_{(C)}$ was estimated from the standard deviation of the flat regions in the curves (regions without crystallization). These variations represent the combined contributions of experimental noise and fitting error. The standard deviation of these regions was 0.23%; to be conservative, we doubled this value and report an estimated uncertainty of **±0.5%** for all samples and temperatures.

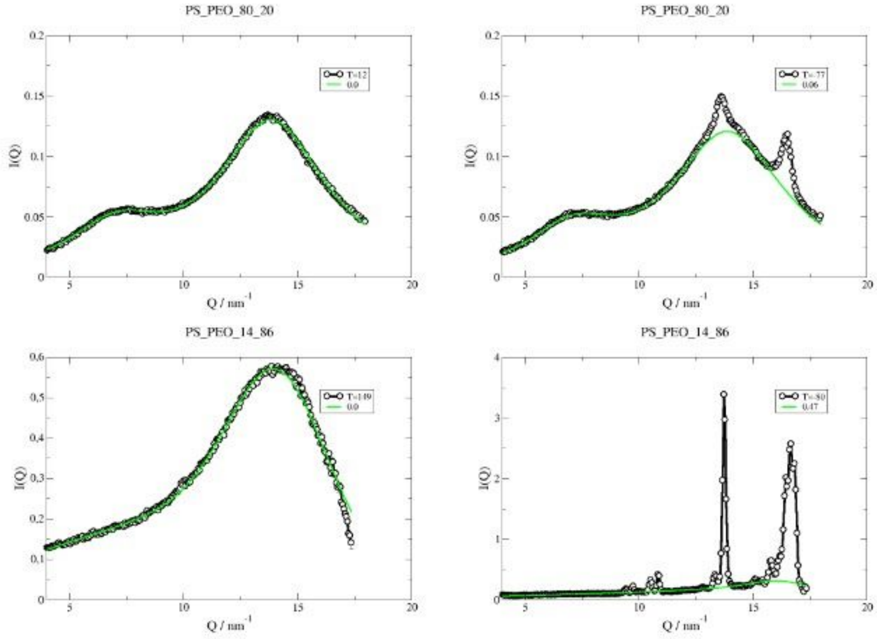

Figure S 1: top: Radially averaged, resampled WAXS data for the sample with the shortest PEO chains in the amorphous (left) and crystalline (right) states. (Bottom) Corresponding data for the sample with the longest PEO chains. Green lines denote fits of the amorphous background.

SAXS data analysis

As described in the text, SAXS data collected at -10°C were fitted for samples showing features consistent with scattering from spherical objects/domains. The simplest appropriate model is the Hard-Sphere model, used here to capture qualitative trends rather than to extract absolute quantitative parameters.

The apparent volume fraction of the non-crystalline cores increased with decreasing PEO content, consistent with the interpretation of two distinct crystallization events. Furthermore, no such spherical-domain scattering was detected in the sample exhibiting only homogeneous crystallization, consistent with the absence of an external crystalline PEO matrix when PS and PEO arms are of comparable length.

The fitting function was:

$$I(q) = scale \cdot P(q) \cdot S_{PY}(q; R^{Hs}, \eta) + bgr + scale_2 \cdot q^{-porod}$$

where *P(q)* is the **form factor of a homogeneous sphere**

$$F(q;R) = \frac{3[\sin(qR) - qR\cos(qR)]}{(qR)^3},$$

and

$$V = \frac{4}{3}\pi R^3,$$

and t

$P(q) = (\Delta\rho)^2 V^2 |F(q;R)|^2, \Delta\rho = b_c^{SAXS} - b_s^{SAXS}$ ·while $S_{(PY)}(q; R^{Hs}, \eta)$ is the Percus–Yevick hard-sphere structure factor**.**

And the structure facture as $S_{PY}(q; R^{Hs}, \eta)$the Percus–Yevick hard-sphere structure factor.

All fitting parameters were adjustable within defined limits: $0 \leq \eta \leq 0.62$ (volume fraction) and $0 \leq R^{Hs} \leq 20$ nm (hard-sphere radius). A fixed contrast was used for all fits, with the scattering length densities of pure PS and PEO adopted for the core and "solvent," respectively. Because the fits are intended to illustrate qualitative trends rather than provide precise quantitative values, this approximation is sufficient.

The two scale parameters in the model account for the low-q power-law contribution and the mid-q region dominated by the sphere scattering. Although this simple model does not reproduce

all features perfectly, it reliably captures the increase in non-crystalline core volume fraction with decreasing PEO content, supporting the interpretation presented in the main text.

REFERENCES

1. (https://jscatter.readthedocs.io/en/latest/formel.html#jscatter.formel.functions.voigt)
